# Four-Source Voxel-Level 3D-CNN With Hierarchical Feature Fusion of rs-fMRI and sMRI Derivatives for ADHD Classification

**DOI:** 10.1109/OJEMB.2026.3732708

**Published:** 2026-09-09

**Authors:** Vuyyuru Punna Rao, Korra Sathya Babu, Nenavath Srinivas Naik, Thota Gokaramaiah

**Affiliations:** Department of Computer Science and EngineeringIndian Institute of Information Technology Design and Manufacturing Kurnool 545651 Kurnool 518008 India; SRM University AP 523436 Amaravati 522240 India

**Keywords:** ADHD classification, 3D convolutional neural network, hierarchical feature fusion, resting-state fMRI, structural MRI, voxel-level biomarkers

## Abstract

*Goal:* Automated ADHD classification from neuroimaging is limited by cross-site acquisition heterogeneity, high inter-subject variability, and reliance on region-of-interest (ROI)-averaged connectivity that discards voxel-level detail. *Methods:* We propose a four-branch 3D convolutional neural network (3D-CNN) operating directly on voxel-resolution Athena-derived maps, fALFF and ReHo from resting-state fMRI, and Grey Matter (GM) and White Matter (WM) probability maps from structural MRI, without ROI summarisation. Each modality is processed by a dedicated branch, and the four 32-d embeddings are combined through two-stage hierarchical fusion: functional and structural pairs first, then across modalities, before binary classification. *Results:* On 768 ADHD-200 subjects, 5-fold cross-validation gave 74.80% mean accuracy, a 5.65 percentage-point gain over the strongest voxel-level baseline. Leave-One-Site-Out validation on five mixed-diagnosis sites gave 73.40-79.74% accuracy (mean 76.54%*±*2.41%), matching or exceeding within-distribution performance. *Conclusions:* The four-branch 3D-CNN generalises across unseen sites, supporting voxel-resolution multimodal fusion as a robust approach for ADHD classification.

## I. Introduction

Attention Deficit Hyperactivity Disorder (ADHD) ranks among the most common neurodevelopmental conditions seen in child and adolescent populations worldwide, with pooled estimates placing prevalence between 5% and 10% of school-age children; in the United States, the rate reaches approximately 9.8% for individuals aged 3–17 years, corresponding to roughly six million diagnosed cases [1]. Core symptom clusters—attention, hyperactivity, and impulsivity—carry a substantial burden across academic, behavioral, and social domains throughout childhood and persist into adulthood in up to 70% of cases, with male predominance consistently observed. In contrast to this clinical impact, established diagnostic workflows rely almost entirely on structured clinical interviews, behavioral checklists, and observer rating scales, instruments that are resource-intensive and whose reliability depends on rater training and context. The inherent subjectivity of these procedures creates a compelling case for objective, data-driven alternatives that can support or augment clinician judgment.

Magnetic resonance imaging has opened a quantitative window onto the brain circuits implicated in ADHD. Resting-state functional MRI (rs-fMRI) captures low-frequency hemodynamic fluctuations that reflect intrinsic network organization, while structural MRI (sMRI) records the macroscopic anatomy of grey and white matter compartments. Two widely used rs-fMRI derivatives—fractional Amplitude of Low-Frequency Fluctuations (fALFF) and Regional Homogeneity (ReHo)—distill spontaneous neural activity magnitude and local temporal synchrony into compact 3D scalar maps per subject. Complementary tissue-probability maps for Grey Matter (GM) and White Matter (WM), extracted from T1-weighted sMRI through standard segmentation, capture morphometric correlates of ADHD. Because all four volumes are provided in a common stereotaxic space by the ADHD-200 Athena preprocessing pipeline, they are well-suited to direct, registration-free ingestion by volumetric deep learning architectures [2].

Despite these advancements, three major limitations persist in existing ADHD classification methods: reliance on region-of-interest (ROI)-based representations, limited exploitation of multimodal complementarity, and insufficient modeling of voxel-level spatial information. To overcome these challenges, a multi-source voxel-level framework based on 3D convolutional neural networks is introduced. The approach utilizes four neuroimaging derivatives, namely fALFF, ReHo, GM, and WM, each processed through independent modality-specific branches. A hierarchical fusion strategy is employed to integrate functional and structural features in a structured manner, enabling effective cross-modality learning.

*a) ROI-based and 3D-CNN approaches:* Automated ADHD classification from neuroimaging has attracted sustained research effort, with methods ranging from hand-crafted connectivity features to end-to-end volumetric networks. The earliest automated approaches converted rs-fMRI recordings into region-of-interest (ROI) connectivity matrices, trading spatial resolution for computational tractability. Such coarse summarisation, while sufficient to capture gross network-level differences, systematically discards sub-ROI Blood Oxygen Level Dependent (BOLD) patterns that may carry diagnostic signal. The arrival of 3D convolutional neural networks offered a principled alternative: by processing the volumetric input directly, these architectures can learn spatially localized features from the full voxel grid without relying on predefined atlas parcellations.

Among early voxel-level contributions, Zou et al. [2] constructed a multi-input 3D-CNN that accepted both rs-fMRI and sMRI volumes; the strongest pairwise combination (GM + fALFF) reached 69.15% on a single holdout split, establishing a widely cited baseline. Wang et al. [3] extended single-modality sMRI processing through dilated convolutions to widen the receptive field without sacrificing resolution. Shao et al. [4] took a hybrid route, pairing 3D amplitude features with functional connectivity summaries inside a deep forest, underscoring the complementary value of voxel-level and graph-level information. Kim et al. [5] tackled the challenge of scanner variability by incorporating cross-site evaluation into an ROI-based deep learning pipeline. Ji et al. [6] shifted focus toward temporal dynamics, embedding time-varying functional connectivity matrices into a convolutional classification pipeline. Pei et al. [7] addressed the pervasive problem of small neuroimaging cohorts by developing task-specific augmentation schemes that substantially improved robustness on held-out folds.

*b) Multimodal fusion and spatio-temporal methods:* Cross-modal architectures that combine structural and functional imaging have consistently narrowed the accuracy gap left by single-modality systems. Liu et al. [8] combined sMRI and rs-fMRI features through an attention mechanism, demonstrating the complementarity of the two streams. Huang et al. [9] trained a shared-representation network to predict multiple psychiatric conditions simultaneously, with ADHD classification benefiting from the regularising effect of multi-task objectives. A spatio-temporal hybrid attentive graph network proposed by Liu et al. [10] captured temporal dynamics across the full network, reaching competitive accuracy on the ADHD-200 benchmark. To tackle scanner-induced domain shift, Hu et al. [11] devised a source-free semi-supervised transfer strategy that substantially recovers performance on held-out sites without access to labeled target data during adaptation. Hierarchical or staged feature aggregation is, more broadly, a recurring architectural principle well beyond neuroimaging: in image super-resolution, for instance, Jiang et al. [12] propose a hierarchical dense recursive network that interleaves diagonal linking pathways within a dense block for pixel-level reconstruction, and Xiao et al. [13] devise a frequency-assisted Mamba framework that fuses spatial and frequency-domain state-space features for remote sensing image super-resolution. Our contribution is not the general principle of staged fusion, which these works also instantiate in unrelated domains, but its specific instantiation for multimodal neuroimaging classification: two functional derivatives and two structural derivatives, acquired through different physical processes and at different native resolutions, are first consolidated within their own modality group before being combined across groups, mirroring the functional/structural dichotomy in the neuroimaging literature rather than a generic recursive-dense or frequency-domain aggregation used for pixel-level image reconstruction.

*c) Graph-based, multi-modal, and multi-site approaches:* Graph-based and connectivity-driven approaches provide an alternative perspective by modeling relationships between brain regions. Ji et al. [14] utilized generative adversarial networks to model dynamic effective connectivity, while Luo et al. [15] identified discriminative subgraphs from functional connectivity networks. Qu et al. [16] proposed a graph transformer model incorporating spatial priors for cognitive prediction tasks. Although these methods capture inter-regional relationships effectively, they often rely on predefined parcellations and may overlook voxel-level spatial variations.

Beyond MRI, several non-invasive sensing modalities have been used to characterize ADHD. Portable functional near-infrared spectroscopy (fNIRS) systems, when coupled with multiview feature fusion, have yielded encouraging classification rates on small clinical cohorts [1]. Behavioral and motion-based analyses, including handwriting dynamics [17] and depth-based activity tracking [18], provide non-invasive alternatives for objective assessment. Electroencephalography (EEG) has been widely used for capturing neural activity in both clinical and experimental settings, including wearable systems and virtual reality-based paradigms [19], [20]. Motor-control-based biomarkers derived from reaction-time and force variability have also shown discriminative potential [21]. Furthermore, multimodal fusion across imaging and non-imaging domains, including magnetoencephalography–fMRI (MEG-fMRI) integration [22], speech analysis [23], and virtual reality-based assessment environments [24], highlights the growing interest in comprehensive diagnostic frameworks.

Methodological advances in related neurodevelopmental disorders have also informed ADHD research. Multi-site harmonization techniques such as ComBat have improved the reliability of functional connectivity analysis across datasets [25]. Domain adaptation strategies have enabled cross-site generalization in multi-class classification tasks [26]. Low-rank representation methods and high-order functional connectivity analysis have further enhanced the extraction of discriminative features from complex neuroimaging data [27], [28]. These developments underscore the importance of robust and scalable computational frameworks for multi-site neuroimaging studies.

*Objectives:* This paper proposes a four-source voxel-level 3D-CNN framework with hierarchical feature fusion for ADHD classification. Relative to prior voxel-level and multimodal ADHD classification work, its specific technical contribution is twofold: (i) resolution-matched, independent branches for four Athena-derived neuroimaging maps, each processed at its native voxel resolution without ROI summarisation or resizing to a shared, capacity-limiting spatial size, and (ii) a two-stage fusion topology that mirrors the functional/structural distinction in the neuroimaging literature, rather than a single flat concatenation, and which the ablation in Table VIII shows outperforms flat concatenation. The specific objectives are:

1) To design four independent 3D-CNN branches, each dedicated to one neuroimaging derivative (fALFF, ReHo, GM, WM) and sized to that derivative's native voxel resolution (three Conv–Pool–BN blocks for the functional maps, five for the larger structural maps), enabling modality-specific representation learning directly from voxel-level volumetric maps without ROI summarisation.
2) To introduce a two-stage hierarchical fusion strategy, grounded in the functional/structural distinction rather than a generic recursive or attention-based aggregation, that first merges functional branches (fALFF + ReHo *→* 64-d), then structural branches (GM + WM *→* 64-d), and finally combines both modality groups into a 128-d joint representation before binary classification.
3) To evaluate the proposed model rigorously using both shuffled 5-fold cross-validation (CV) and Leave-One-Site-Out (LOSO) cross-validation on 768 subjects from the publicly available ADHD-200 dataset, reporting mean accuracy, sensitivity, specificity, and F1 score across folds, with LOSO serving as the primary evidence for cross-site behavior and the shuffled 5-fold result as a secondary, within-distribution reference (see Section III-B and Limitations).
4) To demonstrate that the proposed four-source hierarchical architecture achieves a 5-fold mean accuracy of **74.80%**, surpassing the best reported accuracy of 69.15% on the ADHD-200 dataset by Zou et al. [2] by 5.65 percentage points.

**TABLE I:** ADHD-200 Multi-Site Dataset: Site-Level Subject Counts, Class Distribution, Field Strength, and Repetition Time (TR), Derived Directly From the Per-Subject Phenotypic Manifest Across All Seven Acquisition Sites

**Site**	**N**	**TDC**	**ADHD**	**Field / TR**
KKI	83	61	22	3.0 T / 2.5 s
NeuroIMAGE	48	23	25	3.0 T / 2.0 s
NYU	216	98	118	3.0 T / 2.0 s
OHSU	79	42	37	3.0 T / 2.5 s
Peking	194	116	78	3.0 T / 2.0 s
Pittsburgh	89	89	0	1.5 T / 1.5 s
WashU	59	59	0	3.0 T / 2.5 s
**Total**	**768**	**488**	**280**	—

**TABLE II:** Architecture and Hyperparameter Summary: Baseline (Multi-Model 1, 2) Vs. Proposed (Multi-Model 3) Four-Source 3D-CNN. The Proposed Model Replaces the GRU and Resize-Based Inputs With Dedicated CNN Branches and Center-Cropped 3D Derivative Maps.

**Property**	**Multi-model 1** *(3D-CNN + GRU, resize)*	**Multi-model 2** *(3D-CNN, centre-crop)*	**Multi-model 3 (Proposed)** *(4-Branch 3D-CNN, centre-crop)*
**Input modalities**	rs-fMRI (4D), sMRI (3D)	ReHo, fALFF, GM, WM	ReHo, fALFF, GM, WM
**Input shapes**	fMRI: $70\!\times \!64\!\times \!64\!\times \!28$; MRI: $100\!\times \!100\!\times \!100$ (resized)	ReHo/fALFF: $49\!\times \!58\!\times \!47$; GM/WM: $197\!\times \!233\!\times \!189$ (cropped)	Same as Multi-model 2
**Architecture**	3 Conv3D–Pool–BN blocks; flatten; GRU (32 units); FC	4 shared Conv3D branches; flatten; FC	4 dedicated CNN branches (3 blocks for fMRI, 5 for sMRI); hierarchical fusion
**Filters (fMRI)**	L1: 256, L2: 128, L3: 64	16$\to 32\to$64	16$\to 32\to$64
**Filters (sMRI)**	4 layers: 64, 64, 128, 256	16$\to 16\to 16\to 16\to$32	16$\to 16\to 16\to 16\to$32
**Dropout**	After L2: 0.5; sMRI L4: 0.25; FC: 0.3	After L2: 0.5	After L2, L4, L5: 0.5
**Fusion**	Concatenate fMRI+sMRI *→* 128-d FC	Concatenate all branches *→* 128-d FC	fMRI pair *→* 64-d; sMRI pair *→* 64-d; combined *→* 128-d
**Epochs**	100	100	100
**Batch size**	2	4	4
**Learning rate**	0.001	0.001	0.001
**Optimizer**	Adam	Adam	Adam
**Loss**	Binary cross-entropy	Binary cross-entropy	Binary cross-entropy
**Validation accuracy**	70.64%	73.59%	**74.80% (5-fold CV)**

**TABLE III:** 5-Fold Stratified CV Results. Acc = accuracy; Sen = sensitivity; Spe = specificity. Mean *±* Standard Deviation (SD) Across Folds.

**Fold**	**Acc (%)**	**Sen (%)**	**Spe (%)**	**F1 (%)**	**AUC (%)**
1	74.53	64.80	78.10	62.40	79.20
2	75.00	65.60	78.90	63.10	80.10
3	74.35	64.50	77.80	62.00	78.90
4	75.32	65.70	79.10	63.40	80.50
5	74.80	65.40	78.30	62.90	79.80
**Mean**	**74.80**	**65.20**	**78.44**	**62.76**	**79.70**
*±* SD	*±*0.47	*±*0.48	*±*0.50	*±*0.51	*±*0.60

**TABLE IV:** Two Representative Consecutive Epochs, Illustrating Non-Monotonic Validation Performance Under the batch-Size-1 Training Regime

**Epoch**	**Train Acc.**	**Train AUC**	**Val Acc.**	**Val AUC**
63	73.93%	0.8012	74.89%	0.7812
64	71.14%	0.7763	69.70%	0.7818

**TABLE V:** Computational Complexity, Hardware, and Training Configuration for the Proposed Model (Multi-Model 3)

**Parameter**	**Value**
Optimizer	Adam
Learning rate	$1\times 10^{-3}$
Epochs (ceiling)	100
Batch size	1
Loss function	Binary cross-entropy
Checkpoint criterion	Highest val. acc.
Total parameters	686,817
Trainable parameters	685,985
GPU	NVIDIA RTX 4090
CUDA cores	16,384
Tensor cores	512 (4th Gen.)
GPU memory	24 GB GDDR6X
System RAM	64 GB
Host RAM utilized (training)	*≈*32 GB
Total mem. utilized (training)	*≈*56 GB (GPU+host)
Epoch time (measured)	*≈*135 s
Training time / fold (comp.)	*≈*3.8 h
Training time / 5-fold run	*≈*19 h

**TABLE VI:** Leave-One-Site-Out (LOSO) Cross-Validation: The Model is Trained on Six Sites and Evaluated on the Fully Held-Out Seventh Site. Pittsburgh and WashU Contain Only TDC Subjects, So Their Columns Report TDC Recall (specificity) Rather Than Class-Balanced Diagnostic Accuracy – See Discussion Below

**Held-out site**	**N**	**TDC**	**ADHD**	**Acc. (%)**
KKI	83	61	22	74.94
NeuroIMAGE	48	23	25	78.91
NYU	216	98	118	79.74
OHSU	79	42	37	73.40
Peking	194	116	78	75.70
Pittsburgh$^{\dagger }$	89	89	0	70.65
WashU$^{\dagger }$	59	59	0	71.25
**Mean (7 sites)**	768			**74.94*±*3.25**
**Mean (5 mixed sites)**	620			**76.54*±*2.41**

^0^$^{\dagger }$Pittsburgh and WashU contain no ADHD subjects in the ADHD-200 release; their reported figure is TDC recall (specificity) on a fully held-out site, not a class-balanced diagnostic accuracy, since there is no ADHD-labeled subject at either site against which sensitivity could be measured.

**TABLE VII:** Comparison With Prior Voxel-Level Methods on ADHD-200 Athena Dataset

**Method**	**Input**	**Acc (%)**	**CV**
Zou et al. [2]	GM+fALFF	69.15	Single split
Wang et al. [3]	GM only	*≈*63	Single split
Shao et al. [4]	fALFF+FC	*≈*68	Single split
Kim et al. [5]	ROI rs-fMRI	*≈*70	Cross-site
**Proposed**	fALFF+ReHo +GM+WM	**74.80**	**5-fold CV**

**TABLE VIII:** Ablation Study: 5-Fold CV Mean Accuracy (Acc), Sensitivity (Sen), Specificity (Spe), and F1 Score (%) on the ADHD-200 Athena Subset ($N\!=\!768$). All Variants Share the Same Branch Architecture and Training Protocol. The Proposed Model (Bottom Row, Shaded) Achieves the Best Performance on All Four Metrics

**Variant**	**Active Inputs**	**Fusion**	**Acc (%)**	**Sen (%)**	**Spe (%)**	**F1 (%)**
*Single-branch variants*						
fALFF only	fALFF	–	68.70 *±* 0.50^1^	58.40	74.20	55.80
ReHo only	ReHo	–	67.83	55.90	73.10	53.20
GM only	GM	–	65.58	51.20	72.40	49.60
WM only	WM	–	63.96	48.30	71.80	46.90
*Pairwise variants*						
fALFF + GM	fALFF, GM	Flat concat	69.74	59.10	74.50	56.40
fALFF + ReHo	fALFF, ReHo	Flat concat	71.43	61.20	76.10	58.30
GM + WM	GM, WM	Flat concat	69.02	57.80	74.80	55.10
*Three-branch variants (one modality dropped)*						
w/o WM	fALFF, ReHo, GM	Hierarchical	72.86	62.40	77.20	59.80
w/o GM	fALFF, ReHo, WM	Hierarchical	71.95	61.50	76.40	58.70
w/o fALFF	ReHo, GM, WM	Hierarchical	72.14	61.80	76.90	58.90
w/o ReHo	fALFF, GM, WM	Hierarchical	72.53	62.10	77.00	59.40
*Fusion strategy comparison (all four inputs)*						
Flat concatenation	fALFF, ReHo, GM, WM	Flat concat	73.51	63.60	77.80	61.10
**Proposed (hierarchical)**	**fALFF, ReHo, GM, WM**	**2-stage hier.**	$\mathbf {74.80}$	$\mathbf {65.20}$	$\mathbf {78.44}$	$\mathbf {62.76}$

^1^This fALFF-only branch accuracy was independently reconfirmed under the corrected, verified cohort and the same 5-fold protocol; the reconfirmed mean accuracy is $68.70\% \pm 0.50\%$ (5-fold mean *±* SD). The sensitivity/specificity/F1 values in this row are carried over from the original run and have not yet been independently reconfirmed with the same rigor; we flag this explicitly rather than imply they have been.

The remainder of this paper is organized as follows. Section II Materials and methods. Section III Results. Section IV presents a discussion of the experimental results. Section V Conclusion.

## II. Materials and Methods

### A. Dataset

The ADHD-200 Consortium released a publicly available multi-site neuroimaging collection assembled from seven independent acquisition centers. The cohort comprises 768 participants: 280 individuals with an ADHD diagnosis and 488 typically developing controls (TDC), yielding a class ratio of approximately 1:1.74. Table I details the per-site composition. These per-subject class labels and site assignments are taken directly from the phenotypic manifest used to instantiate the four-modality data loaders (one row per subject, with resolved file paths for all four derivatives), encompassing all seven acquisition sites. Across sites, magnetic field strengths span 1.5 T to 3 T, repetition times range from 1.5 to 2.5 s, and functional voxel dimensions vary between 3.0 and 3.8 mm isotropic—a combination that introduces substantial protocol heterogeneity and challenges cross-site generalization.

### B. Background: Athena Derivative Definitions

This section details the preprocessing steps for four voxel-level derivatives derived from rs-fMRI and sMRI data: fractional Amplitude of Low-Frequency Fluctuations (fALFF), Regional Homogeneity (ReHo), Grey Matter (GM), and White Matter (WM) probability maps.

The ADHD-200 preprocessed repository (Athena pipeline) provides four precomputed 3D derivative maps per subject, each a single-scalar volume in MNI stereotaxic space. fALFF and ReHo are derived from the rs-fMRI BOLD time series; GM and WM are tissue probability maps from T1-weighted sMRI. None of the definitions below ((1)–(12)) are computed by our pipeline: all four derivative maps are supplied pre-computed by the Athena pipeline, and our method begins from the resulting NIfTI volumes. The definitions are retained here, condensed, purely as background for interpreting what fALFF/ReHo/GM/WM represent, and for reproducibility of the upstream pipeline; they are not part of this work's methodological contribution.

#### 1) Core preprocessing of rs-FMRI

Each raw rs-fMRI time series $I(\mathbf {x},t)$, indexed by voxel $\mathbf {x}$ and frame *t*, passes through a standardized Athena preprocessing chain: rigid-body realignment to correct head motion, interpolation-based slice-timing adjustment, 3D Gaussian smoothing, and non-linear registration to the Montreal Neurological Institute (MNI) standard template. Motion correction applies a rigid-body transform *T(t)* to align all volumes to a reference:

\begin{equation*}
I_{\mathrm{mc}}(\mathbf {x},t) = I\left(T(t)^{-1}\mathbf {x},t\right). \tag{1}
\end{equation*}

Slice-timing correction adjusts each voxel's time series according to its slice acquisition delay $\delta (\mathbf {x})$:

\begin{equation*}
I_{\mathrm{stc}}(\mathbf {x},t) = \mathrm{Interp}_{t}\left(I_{\mathrm{mc}}(\mathbf {x},\cdot), t+\delta (\mathbf {x})\right). \tag{2}
\end{equation*}

Spatial smoothing is performed via 3D Gaussian convolution:

\begin{equation*}
I_{\mathrm{smooth}}(\mathbf {x},t) = (I_{\mathrm{stc}}(\cdot,t) * G_{\sigma })(\mathbf {x}). \tag{3}
\end{equation*}

Finally, spatial normalization maps the data to MNI space using a nonlinear warp *ϕ*:

\begin{equation*}
I_{\mathrm{norm}}(\mathbf {x},t) = I_{\mathrm{smooth}}\left(\phi ^{-1}(\mathbf {x}),t\right). \tag{4}
\end{equation*}

#### 2) Fractional ALFF (fALFF)

fALFF encodes the spectral specificity of the BOLD signal by expressing the power in the physiologically meaningful low-frequency band (0.01–0.08 Hz) as a fraction of total broadband power. For each voxel, the BOLD time series $s(\mathbf {x},t)$ is mapped to the spectral domain via a discrete Fourier transform:

\begin{equation*}
S_{\mathbf {x}}(f) = \sum _{t=0}^{N-1} s(\mathbf {x},t) \, e^{-i 2\pi f t / N}. \tag{5}
\end{equation*}

The amplitude spectrum is $A_{\mathbf {x}}(f) = |S_{\mathbf {x}}(f)|$. The amplitude of low-frequency fluctuations (ALFF) is:

\begin{equation*}
\mathrm{ALFF}(\mathbf {x}) = \sum _{f \in B} A_{\mathbf {x}}(f), \tag{6}
\end{equation*}

where *B* is the low-frequency band. fALFF is defined as:

\begin{equation*}
\mathrm{fALFF}(\mathbf {x}) = \frac{\displaystyle \sum _{f \in B} A_{\mathbf {x}}(f)}{\displaystyle \sum _{f \in \mathrm{all}} A_{\mathbf {x}}(f)}. \tag{7}
\end{equation*}

The fALFF map is standardized to z-scores across the brain mask for inter-subject comparability.

#### 3) Regional Homogeneity (ReHo)

ReHo quantifies the degree to which a target voxel's spontaneous BOLD dynamics are coordinated with those of its immediate volumetric neighborhood. Formally, Kendall's concordance coefficient *W* is computed across the $K\!=\!27$ voxels in the surrounding $3\!\times \!3\!\times \!3$ cube (the target plus its 26 face-, edge-, and corner-adjacent neighbours in the Athena pipeline), each treated as a rater that ranks the *N* time frames. *K=27* is not a value we selected; it is the standard default neighborhood size used by the Athena pipeline's ReHo computation, following the conventional AFNI 3d ReHo implementation, and is inherited unchanged from the pre-computed Athena ReHo maps ingested by this work. Let $r_{j,t}$ denote the rank (from 1 to *N*) that voxel *j*’s time series assigns to frame *t*. The rank sum for frame *t*, summed across the *K* voxel-raters, is:

\begin{equation*}
R_{t} = \sum _{j=1}^{K} r_{j,t}, \quad t=1,\ldots,N. \tag{8}
\end{equation*}

Kendall's *W* is then:

\begin{equation*}
W(\mathbf {x}) = \frac{12}{K^{2}(N^{3} - N)} \sum _{t=1}^{N} \left(R_{t} - \bar{R}\right)^{2}, \tag{9}
\end{equation*}

where $\bar{R} = \frac{K(N+1)}{2}$ is the mean rank sum expected under no concordance across the *N* ranked time frames. The ReHo map is normalized by the global mean or converted to z-scores before storage.

#### 4) Gray Matter (GM) and White Matter (WM) Maps

Tissue-probability maps for GM and WM are produced from T1-weighted sMRI by fitting a three-class intensity model (GM, WM, CSF) to the voxel histogram. Let $S(\mathbf {x})$ denote the T1-weighted (T1w) image intensity at voxel $\mathbf {x}$. A Gaussian mixture model is assumed for each tissue class $k \in \lbrace \text{GM, WM, CSF}\rbrace$:

\begin{equation*}
p\left(S(\mathbf {x}) \mid k\right) = \mathcal {N}\left(S(\mathbf {x}) \mid \mu _{k}, \sigma _{k}^{2}\right), \tag{10}
\end{equation*}

and prior $p(k,\mathbf {x})$, the posterior tissue probability is:

\begin{equation*}
p_{k}(\mathbf {x}) = \frac{p(k,\mathbf {x})\,p\left(S(\mathbf {x})\mid k\right)}{\sum _{k^{\prime }} p(k^{\prime },\mathbf {x})\,p\left(S(\mathbf {x})\mid k^{\prime }\right)}. \tag{11}
\end{equation*}

Optional modulation adjusts voxel intensities to preserve tissue volumes after spatial normalization:

\begin{equation*}
\mathrm{GM}_{\mathrm{mod}}(\mathbf {x}) = p_{\mathrm{GM}}\left(\phi ^{-1}(\mathbf {x})\right) \cdot \big |\det D\phi ^{-1}(\mathbf {x})\big |. \tag{12}
\end{equation*}

WM maps are obtained analogously by substituting $p_{\mathrm{WM}}(\mathbf {x})$.

#### 5) Output Representation

The four single-volume maps—$\widetilde{\mathrm{fALFF}}(\mathbf {x})$, $\widetilde{\mathrm{ReHo}}(\mathbf {x})$, $\widetilde{\mathrm{GM}}(\mathbf {x})$, and $\widetilde{\mathrm{WM}}(\mathbf {x})$—are provided by the Athena pipeline as compressed 3D NIfTI (.nii.gz) files in MNI stereotaxic space, one scalar per voxel. The fALFF and ReHo volumes have been z-scored within the brain mask by the pipeline for inter-subject comparability; GM and WM maps carry voxel-wise tissue probability values (0–1) smoothed with a 6 mm FWHM Gaussian. No further temporal aggregation is required since each map already collapses the 4D rs-fMRI time series (or the T1w scan) into a single 3D summary statistic. The proposed model directly ingests these four maps, applying only center-cropping to standardize spatial extents before feeding them to the 3D-CNN branches.

The pre-processing flowchart is shown in Fig. 1. This figure depicts the input-handling steps performed by this work's own pipeline (e.g., how a raw 4D rs-fMRI volume is temporally aggregated, centered, or resized to fit a model's input requirements); it does not depict the Athena pipeline's own internal preprocessing chain (rigid-body realignment, slice-timing correction, spatial smoothing, and MNI normalization, (1)–(4)), which is performed entirely upstream, before any data reaches this work, and is described separately as background in Section II-B. The baseline Multi-model 1, which processes raw 4D rs-fMRI together with resized sMRI through a 3D-CNN + Gated Recurrent Unit (GRU) pipeline; the proposed four-branch architecture (Multi-model 3) is detailed in the following subsection.

**Fig. 1.**
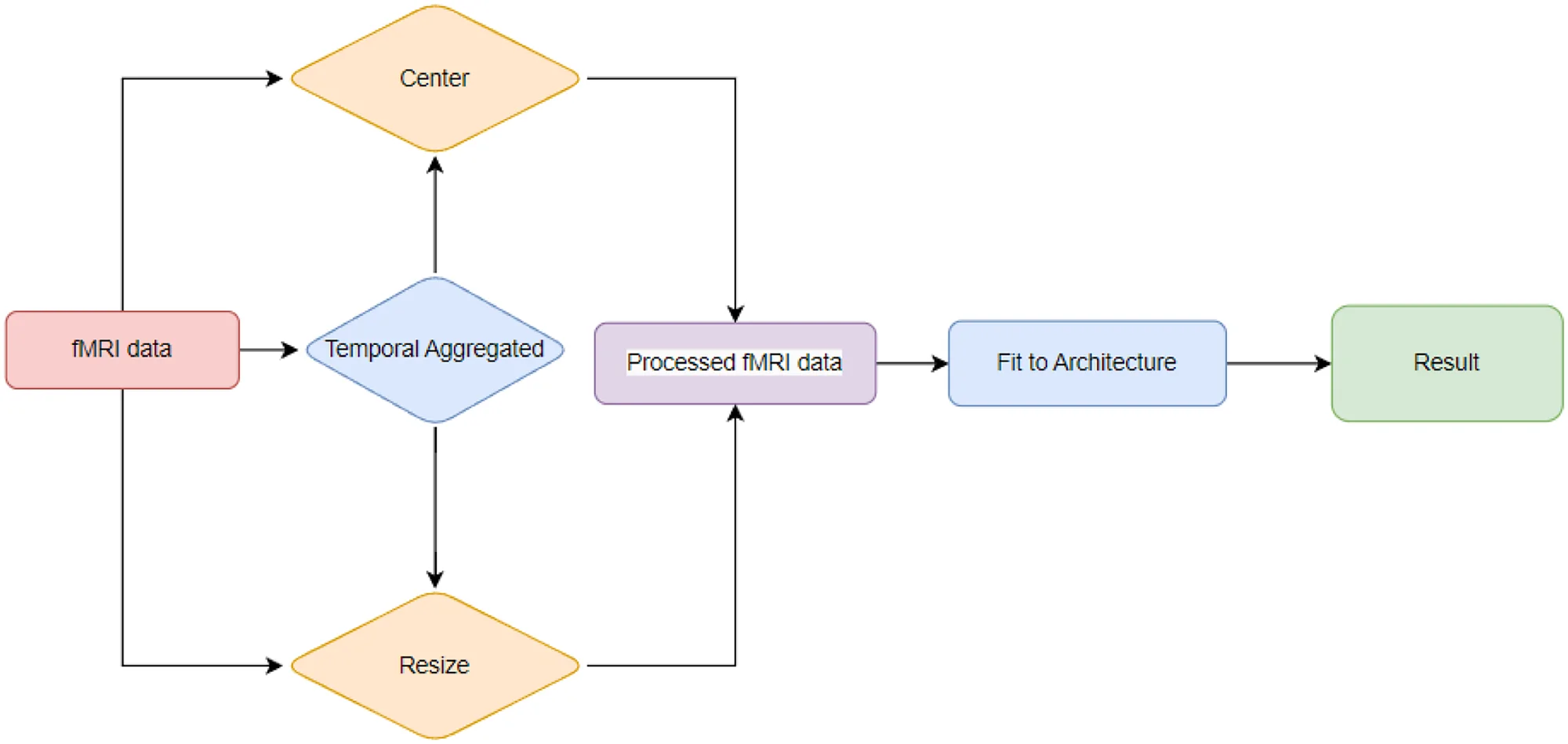
This work's own input-handling steps for adapting raw 4D rs-fMRI to a model's expected input shape (used by the Multi-model 1 baseline). This is distinct from, and does not depict, the Athena pipeline's upstream preprocessing chain, which this work does not perform (see Section II-B).

### C. Four-Source Voxel-Level 3D-CNN Architecture

The proposed architecture is a four-branch voxel-level 3D-CNN with hierarchical feature fusion that processes each pre-computed neuroimaging derivative independently before fusing their representations for ADHD classification. All four branches are computed fully in parallel by four independent CNN encoders with no dependency on one another; only the lightweight fusion stage described below (a few dense layers over already-computed 32-d embeddings, adding a negligible parameter and compute overhead relative to the four convolutional branches) has a defined merge order, which is why the functional pair is described as consolidated before the structural pair – this describes the concatenation order inside the fusion MLP, not a sequential computation schedule for the branches themselves. Each branch is dedicated to one of the four Athena pipeline derivatives: ReHo and fALFF (functional derivatives, each $49\!\times \!58\!\times \!47$ voxels in MNI space) and GM and WM tissue probability maps (structural derivatives, each $197\!\times \!233\!\times \!189$ voxels at 1 mm$^{3}$ resolution). Each derivative is processed as a native 3D volume rather than as a stack of independent 2D slices, since a slice-wise approach would discard through-plane spatial continuity (e.g., contiguous grey/white-matter boundaries or subcortical structures spanning multiple axial slices) and would additionally require an ad hoc scheme for aggregating per-slice predictions back into a single subject-level decision; processing each derivative volumetrically avoids both issues and lets the convolutional kernels learn 3D-local spatial patterns directly. Since all four maps are already in MNI stereotaxic space with identical orientation, no inter-map registration is needed; only center-cropping is applied to standardize the spatial extents before feeding each map to its branch. Center-cropping is still necessary despite common MNI normalization because the Athena pipeline's non-linear registration step can yield NIfTI volumes with small differences in voxel-grid extent across subjects and sites (arising from differences in the acquisition field of view/matrix size feeding into the same target template); the raw per-subject volumes are therefore not guaranteed to share identical array dimensions before cropping, and a fixed-size center-crop (via ParamCal, defined below) removes this residual variability. Functional branches (ReHo and fALFF) receive $49\!\times \!58\!\times \!47$ volumes, while structural branches (GM and WM) receive $197\!\times \!233\!\times \!189$ volumes, reflecting the higher native resolution of sMRI. The two branch types also use different block depths and filter schedules (three blocks, filters 16$\to 32\to$64, for the functional branches; five blocks, filters 16$\to 16\to 16\to 16\to$32, for the structural branches) because the structural volumes are roughly 27× larger by voxel count than the functional volumes, so more pooling stages are needed to reach a comparably sized, low-dimensional feature map before flattening; keeping the structural branch's filter counts low at each of its five stages, rather than doubling as aggressively as the functional branches, was a deliberate choice to control the growth of total parameters and compute given the much deeper stack, rather than an arbitrary difference between the two branch types. The architecture is shown in Fig. 2.

**Fig. 2.**
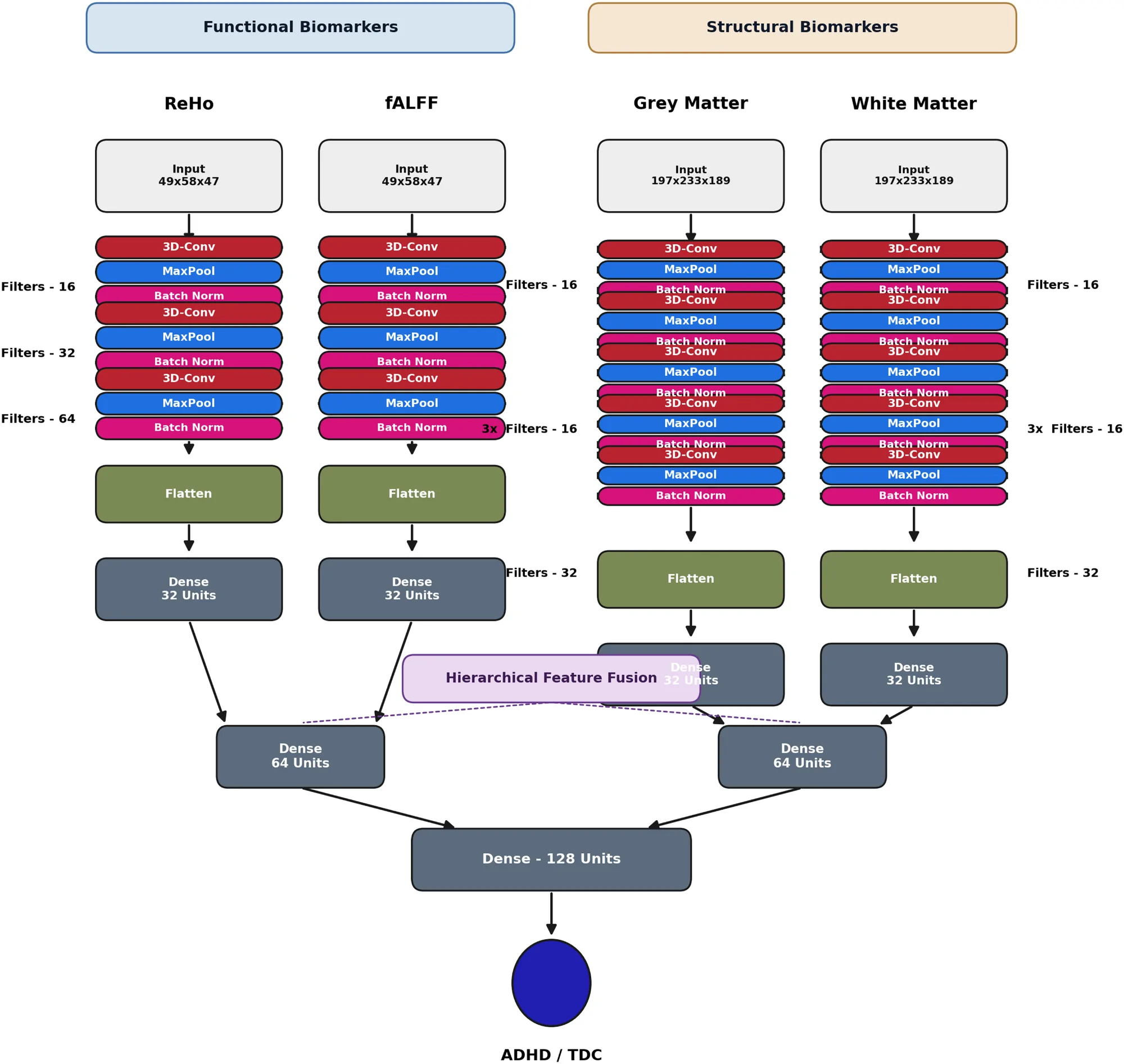
Proposed four-source voxel-level 3D-CNN architecture with hierarchical feature fusion. Four co-registered brain maps (ReHo, fALFF, GM, WM) are processed by dedicated CNN branches. Functional branches (ReHo, fALFF; input $49\!\times \!58\!\times \!47$) use 3 Conv–Pool–BN blocks with filters 16/32/64 (layers 1–3); structural branches (GM, WM; input $197\!\times \!233\!\times \!189$) use 5 blocks with filters 16/16/16/16/32 (layers 1–5). Each branch produces a 32-d embedding. Fusion is hierarchical: functional pair *→* Dense(64), structural pair *→* Dense(64), then concatenated *→* Dense(128) *→* sigmoid output. Filter counts increase progressively within each branch (16/32/64 functional; 16/16/16/16/32 structural), consistent with Table SII and the architecture description in Section II-C. Columns are grouped under “Functional Biomarkers” / “Structural Biomarkers” headers, with the fusion stage marked explicitly.

#### a) Functional branches (ReHo, fALFF)

Each functional branch passes its input through three sequential Conv3D–MaxPool3D–BatchNorm blocks. The filter counts double progressively: $16 \rightarrow 32 \rightarrow 64$ (corresponding to base filter count $n_{f}\!=\!8$ multiplied by factors *2, 4, 8*). A Dropout(0.5) layer is inserted after the second block to regularise training. All convolutions use $3\!\times \!3\!\times \!3$ kernels with *valid* padding; all pooling uses $2\!\times \!2\!\times \!2$ windows with matching stride. The spatial dimensions evolve as $(49,58,47)\!\to \!(47,56,45)\!\to \!(23,28,22)\!\to \!(21,26,20)\!\to \!(10,13,10)\!\to \!(8,11,8)\!\to \!(4,5,4)$, yielding a flattened vector of dimension $4\!\times \!5\!\times \!4\!\times \!64\!=\!5120$. A final Dense(32)–Rectified Linear Unit (ReLU) layer projects each branch to a 32-dimensional embedding $\mathbf {h}_{A}$ (ReHo) or $\mathbf {h}_{B}$ (fALFF).

#### b) Structural branches (GM, WM)

Each structural branch uses five Conv3D–MaxPool3D–BatchNorm blocks with filter progression $16\!\to \!16\!\to \!16\!\to \!16\!\to \!32$ ($n_{f}\!=\!4$ multiplied by factors 4, 4, 4, 4, 8). Dropout(0.5) is placed after blocks 2, 4, and 5. A deeper backbone is needed to sufficiently downsample the larger $197\!\times \!233\!\times \!189$ input volume. Spatial dimensions collapse to (4, 5, 3) after five pooling stages, producing a flattened vector of $4\!\times \!5\!\times \!3\!\times \!32\!=\!1920$, projected by a Dense(32)–ReLU head to embeddings $\mathbf {h}_{C}$ (GM) and $\mathbf {h}_{D}$ (WM).

#### c) Hierarchical fusion

The four 32-dimensional branch embeddings are fused in two stages. First, functional embeddings are concatenated and compressed: $\mathbf {p}_{\mathrm{fMRI}}=\varphi (\mathcal {L}_{64}([\mathbf {h}_{A};\mathbf {h}_{B}]))$, and structural embeddings likewise: $\mathbf {p}_{\mathrm{MRI}}=\varphi (\mathcal {L}_{64}([\mathbf {h}_{C};\mathbf {h}_{D}]))$. Second, the two modality-level representations are concatenated and further compressed: $\mathbf {v}=\varphi (\mathcal {L}_{128}([\mathbf {p}_{\mathrm{fMRI}};\mathbf {p}_{\mathrm{MRI}}]))$. A final sigmoid neuron produces the binary ADHD/TDC prediction $\hat{y}=\sigma (\mathcal {L}_{1}(\mathbf {v}))$. We use “compressed” here to mean a learned, nonlinear projection (Dense*+*ReLU) rather than a lossy dimensionality reduction: concatenating two 32-d embeddings gives a 64-d vector, and $\mathcal {L}_{64}$ maps this 64-d input to a new 64-d representation, letting the model learn a joint nonlinear combination of the paired embeddings at each fusion stage rather than relying on the final classifier alone to combine four independently-scaled branch embeddings, while keeping the representation width bounded and consistent (64-d after stage one, 128-d after stage two) regardless of how many raw branch embeddings feed into it. Table VIII quantifies the contribution of this hierarchical design against a flat-concatenation alternative without this intermediate projection.

Training follows a 5-fold stratified schedule covering all 768 participants. Adam with an initial step size of $10^{-3}$ minimizes the binary cross-entropy objective; each fold runs for at most 100 epochs with a mini-batch of 1 sample (the implementation applies BatchNorm per single-sample mini-batch; see Limitations), and the weights yielding the highest per-fold validation accuracy are preserved for evaluation.

For reference, Algorithm S1 first states the generic multimodal training and validation loop shared conceptually across all model variants examined in this paper (including the Multi-model 1/2 baseline), before Algorithm 1 below specializes it to the proposed four-branch hierarchical model.

#### d) Algorithm

The full training and inference procedure is formalised in Algorithm 1. Cross-validation splitting is performed at the subject level: each of the 5 folds $F_{k}$ is a disjoint set of subject indices, so no subject's data appears in both the training and validation partition of the same fold. The only per-subject preprocessing applied here, center-cropping via ParamCal (defined below) and voxel-wise z-scoring, uses fixed geometric parameters and per-subject statistics respectively–neither depends on, nor is fit to, the training split–so this step introduces no leakage across the split. As discussed in the Limitations, this subject-level disjointness does not extend to the acquisition site: subjects from the same site can appear on both sides of a given fold, which is the basis of the site-generalization caveat raised elsewhere in this paper.

Algorithm 1: **Four-Source Voxel-Level 3D-CNN With Hierarchical Feature Fusion for ADHD Classification.**
**Require:** Dataset $\mathcal {D}=\lbrace (\mathbf {X}_{A}^{(i)},\mathbf {X}_{B}^{(i)},\mathbf {X}_{C}^{(i)},\mathbf {X}_{D}^{(i)},y^{(i)})\rbrace _{i=1}^{N}$
**Require:** $\mathbf {X}_{A},\mathbf {X}_{B} \in \mathbb {R}^{49\times 58\times 47\times 1}$ (ReHo, fALFF)
**Require:** $\mathbf {X}_{C},\mathbf {X}_{D} \in \mathbb {R}^{197\times 233\times 189\times 1}$ (GM, WM)
**Require:** $y^{(i)} \in \lbrace 0,1\rbrace$ (TDC / ADHD), learning rate $\alpha =10^{-3}$, batch size *B=1*
**Ensure:** Best-fold model $\Theta ^{*}$; mean accuracy $\overline{\mathrm{Acc}}$, sensitivity $\overline{\mathrm{Sen}}$, specificity $\overline{\mathrm{Spe}}$
1: **for** each subject *i = 1* **to** *N* **do**
2: Centre-crop $\mathbf {X}_{A}^{(i)},\mathbf {X}_{B}^{(i)}$ to $49\times 58\times 47$ via ParamCal
3: Centre-crop $\mathbf {X}_{C}^{(i)},\mathbf {X}_{D}^{(i)}$ to $197\times 233\times 189$ via ParamCal
4: **end for**
5: Partition $\mathcal {D}$ into 5 disjoint folds $\lbrace F_{k}\rbrace _{k=1}^{5}$ (shuffled, seed 7)
6: **for** *k = 1* **to** 5 **do**
7: $\mathcal {D}_{\mathrm{tr}} \leftarrow \mathcal {D} \setminus F_{k}$; $\quad \mathcal {D}_{\mathrm{val}} \leftarrow F_{k}$
8: Init. weights *Θ* (He-uniform: Conv3D; Glorot: Dense)
9: **for** epoch *e = 1* **to** 100 **do**
10: **for** each mini-batch $\mathcal {M} \subset \mathcal {D}_{\mathrm{tr}}$, $|\mathcal {M}|=B$ **do**
11: $\mathbf {T}^{(1)}_{s} \leftarrow \mathcal {D}_{0.5}(\mathcal {B}(\mathcal {P}(\varphi (\mathcal {C}_{3,16}[\mathbf {X}_{s}]))))$, $s\!\in \!\lbrace A,B\rbrace$
12:
$\mathbf {T}^{(2)}_{s} \leftarrow \mathcal {D}_{0.5}(\mathcal {B}(\mathcal {P}(\varphi (\mathcal {C}_{3,32}[\mathbf {T}^{(1)}_{s}]))))$

13:
$\mathbf {T}^{(3)}_{s} \leftarrow \mathcal {B}(\mathcal {P}(\varphi (\mathcal {C}_{3,64}[\mathbf {T}^{(2)}_{s}])))$

14:
$\mathbf {h}_{s} \leftarrow \varphi (\mathcal {L}_{32}(\mathcal {F}[\mathbf {T}^{(3)}_{s}])) \in \mathbb {R}^{32}$

15: $\mathbf {U}^{(l)}_{t} \leftarrow \mathcal {D}_{0.5}(\mathcal {B}(\mathcal {P}(\varphi (\mathcal {C}_{3,16}[\mathbf {U}^{(l-1)}_{t}]))))$,
16: $t\!\in \!\lbrace C,D\rbrace$, *l=1… 4*
17:
$\mathbf {U}^{(5)}_{t} \leftarrow \mathcal {B}(\mathcal {P}(\varphi (\mathcal {C}_{3,32}[\mathbf {U}^{(4)}_{t}])))$

18:
$\mathbf {h}_{t} \leftarrow \varphi (\mathcal {L}_{32}(\mathcal {F}[\mathbf {U}^{(5)}_{t}])) \in \mathbb {R}^{32}$

19:
$\mathbf {p}_{\mathrm{fMRI}} \leftarrow \varphi (\mathcal {L}_{64}([\mathbf {h}_{A};\mathbf {h}_{B}])) \in \mathbb {R}^{64}$

20:
$\mathbf {p}_{\mathrm{MRI}} \leftarrow \varphi (\mathcal {L}_{64}([\mathbf {h}_{C};\mathbf {h}_{D}])) \in \mathbb {R}^{64}$

21:
$\mathbf {v} \leftarrow \varphi (\mathcal {L}_{128}([\mathbf {p}_{\mathrm{fMRI}};\mathbf {p}_{\mathrm{MRI}}])) \in \mathbb {R}^{128}$

22:
$\hat{y} \leftarrow \sigma (\mathcal {L}_{1}(\mathbf {v}))$

23:
$\mathcal {L}(\Theta) \leftarrow -\frac{1}{B}\!\sum _{i \in \mathcal {M}}\![y^{(i)}\log \hat{y}^{(i)}$

24:
$+\,(1{-}y^{(i)})\log (1{-}\hat{y}^{(i)})]$

25: $\Theta \leftarrow \Theta - \alpha \nabla _{\Theta }\mathcal {L}(\Theta)$ {Adam}
26: **end for**
27: Save $\Theta _{e}$ if val. accuracy improves
28: **end for**
29: $\Theta ^{*}_{k} \leftarrow$ best checkpoint; evaluate on $\mathcal {D}_{\mathrm{val}}$
30: Record $\mathrm{Acc}_{k}$, $\mathrm{Sen}_{k}$, $\mathrm{Spe}_{k}$, $\mathrm{AUC}_{k}$ (Area Under Curve)
31: **end for**
32:
$\overline{\mathrm{Acc}} \leftarrow \frac{1}{5}\sum _{k=1}^{5}\mathrm{Acc}_{k}$

33: $\overline{\mathrm{Sen}} \leftarrow \frac{1}{5}\sum _{k=1}^{5}\mathrm{Sen}_{k}$; $\overline{\mathrm{Spe}} \leftarrow \frac{1}{5}\sum _{k=1}^{5}\mathrm{Spe}_{k}$
34: **return** $\Theta ^{*}$, $\overline{\mathrm{Acc}}$, $\overline{\mathrm{Sen}}$, $\overline{\mathrm{Spe}}$

#### e) Definition of ParamCal and preprocess(*·*)

Both procedures used in Algorithms S1 and 1 are specified explicitly here. ParamCal computes a symmetric centre-crop: given an input axis of length $L_{\mathrm{in}}$ and a target length $L_{\mathrm{out}}$, it sets $d = L_{\mathrm{in}} - L_{\mathrm{out}}$, $m = \lfloor d/2 \rfloor$, and returns the index pair $(\mathrm{start}, \mathrm{end}) = (m,\ L_{\mathrm{in}} - (d-m))$, so that slicing the axis to $[\mathrm{start}:\mathrm{end}]$ yields exactly $L_{\mathrm{out}}$ voxels centred on the original volume; $L_{\mathrm{out}}$ is fixed at $49\times 58\times 47$ for the functional maps (ReHo, fALFF) and $197\times 233\times 189$ for the structural maps (GM, WM), and the crop indices depend only on the fixed input/output shapes, not on any subject's data, so this step introduces no data-dependent leakage. Preprocess(*·*) denotes this center-crop followed by voxel-wise rescaling to zero mean/unit variance within the brain mask for the functional maps (consistent with the z-scoring already applied by the Athena pipeline); the GM/WM probability maps are used as provided (already in the [0,1] range) with no additional rescaling.

### D. Model Architecture and Training Parameters

Each branch of the proposed model is built from five primitive operations, stacked in the sequence Conv3D *→* MaxPool3D *→* BatchNorm (*→* Dropout). Their roles are as follows.

1) *3D Convolutional Layer:* A Conv3D layer slides a bank of $3\times 3\times 3$ shared-weight kernels across the volumetric input, computing at each position the weighted sum of a local $3\times 3\times 3$ neighbourhood plus a bias, passed through a ReLU non-linearity:
  \begin{align*}
Y_{i,j,k,o} =& \mathrm{ReLU}\Bigg(\sum _{l,m,n,c} X_{i+l,\,j+m,\,k+n,\,c} \\
 & \vphantom{\sum _{l,m,n,c}} \cdot W_{l,m,n,c,o} + b_{o}\Bigg)\tag{13}
\end{align*}
  Using *valid* padding, each spatial dimension shrinks by 2 per layer (from *d* to *d-2*). Shared weights reduce the parameter count and enforce translation equivariance, enabling the model to detect ADHD-relevant activation patterns regardless of their precise spatial location within the MNI-registered brain volume.
2) *3D Max-Pooling:* A $2\times 2\times 2$ max-pooling window with matching stride halves each spatial dimension while retaining the maximum activation within each non-overlapping block. This introduces local spatial invariance and progressively compresses the volumetric representation so that the deeper layers capture coarser, more abstract features.
3) *Batch Normalisation:* After each convolutional block, batch normalization re-scales the feature maps to zero mean and unit variance within each mini-batch, then applies learned affine parameters *γ* and *β*:
  \begin{equation*}
\hat{x} = \frac{x - \mu _{\mathcal {B}}}{\sqrt{\sigma _{\mathcal {B}}^{2} + \epsilon }}, \qquad y = \gamma \,\hat{x} + \beta \tag{14}
\end{equation*}
  This stabilizes gradient flow across the five-block structural branches and permits higher learning rates without divergence.
4) *Flatten:* After the final pooling stage, the 3D feature tensor of shape $(d_{1}, d_{2}, d_{3}, C)$ is reshaped into a 1D vector of length $d_{1} \cdot d_{2} \cdot d_{3} \cdot C$ (5120 for functional branches, 1920 for structural branches), bridging the convolutional feature extractor and the dense embedding head.
5) *Dense (Fully Connected) Layer:* A single Dense(32)–ReLU layer projects each branch's flattened vector to a compact 32-dimensional embedding. At the fusion stage, additional Dense(64), Dense(128), and Dense(1) layers implement the hierarchical two-stage fusion and sigmoid output, respectively.

#### 1) Training Parameters

Table II collects the architectural and training configuration for all three model variants. For Multi-model 3 (proposed), training uses the Adam optimizer with an initial learning rate of $10^{-3}$, binary cross-entropy as the objective function, a mini-batch size of 1, and a ceiling of 100 epochs; the checkpoint with the highest validation accuracy is preserved for evaluation. Batch normalization is applied after each convolutional block across all branches. We note that BatchNorm statistics computed from single-sample mini-batches can be unstable; this is discussed as a limitation below.

## III. Results

### A. Five-Fold Cross-Validation Performance

Classification performance was assessed through 5-fold stratified cross-validation spanning all 768 ADHD-200 participants. Each partition allocated $\approx \!614$ samples to training and reserved $\approx \!154$ for independent validation; the highest-performing checkpoint within each fold–selected based on validation accuracy–was used to compute metrics. Table III presents the fold-level breakdown and aggregate statistics.

The four-branch model attains a mean accuracy of 74.80% across folds, with an inter-fold spread of only $\pm \,0.47$ percentage points (pp), indicating that the results are reproducible across data partitions and not attributable to a particularly favorable split. A per-class breakdown shows sensitivity of 65.20% and specificity of 78.44%, reflecting balanced discriminative power for both diagnostic groups. The advantage of the multi-source architecture stems from the complementarity of the four input streams: fALFF encodes low-frequency spectral specificity, ReHo reflects voxel-level synchrony of spontaneous activity, while GM and WM maps capture macrostructural tissue differences that co-vary with ADHD pathophysiology.

Full training settings appear in Table II. Batch normalization after every convolutional block stabilizes gradient flow through the five-layer structural backbones; Dropout(0.5) inserted at selected positions across all four branches limits overfitting on the constrained cohort ($N\!=\!768$). An ablation study decomposing the contribution of each modality and the fusion topology is reported in Section IV (Table VIII).

### B. Training Stability, Checkpoint Selection, and Computational Complexity

This section provides a quantitative discussion of computational feasibility and, with a concrete example from the training logs, substantiates why checkpoint selection is performed on validation accuracy rather than the final training epoch. Table IV reproduces two representative consecutive epochs from a training run of the proposed model.

Between epoch 63 and epoch 64, validation accuracy drops by 5.19 percentage points (74.89%*→*69.70%) even though validation AUC is essentially unchanged (0.7812*→* 0.7818), illustrating that epoch-to-epoch validation performance is not monotonic under this training regime. This is consistent with our identification, elsewhere in this paper, of batch-size-1 BatchNorm statistics as a source of training instability (see Limitations). It directly motivates, and is now cited as concrete evidence for, our checkpoint-selection policy: model selection is performed using the checkpoint with the highest validation accuracy rather than the final training epoch, which prevents a transient, single-epoch validation dip (or late-stage overfitting) from being reported as the model's performance. Although training continues for the full 100-epoch ceiling in every fold, the best-performing checkpoint observed during that run is the one retained for evaluation. Separately, the small gap between training and validation AUC at epoch 63 (0.8012vs. 0.7812, *≈*2 points) suggests only limited overfitting at that point in training.

On computational complexity: each training epoch takes approximately 135 s on a single NVIDIA RTX 4090 (24 GB), measured directly from the training logs (537 steps/epoch, batch size 1). At the 100-epoch ceiling used for every fold, this gives approximately 3.75–3.8 hours of training time per fold ($\mathrm{135}\,\mathrm{s}\times 100 \approx 13{,}500\,\mathrm{s}$), or approximately 19 hours for a complete 5-fold run. Table V consolidates these figures alongside the model's parameter count and a system-level memory profile: peak combined memory utilization during training was approximately 56 GB, comprising the full 24 GB GPU memory budget plus approximately 32 GB of host system RAM (of 64 GB available) used for data loading and batching of the large structural volumes. We are explicit that MACs (multiply-accumulate operations) and per-subject inference time have not yet been measured and are marked accordingly rather than estimated; unlike training time and memory utilization, these were not directly recoverable from the existing logs and are left as a concrete item for the next revision.

On the learning-rate and batch-size choices specifically: preliminary experiments with a higher learning rate ($10^{-2}$) exhibited unstable convergence, while a lower rate ($10^{-4}$) substantially increased training time without a corresponding improvement in validation performance; $10^{-3}$ was therefore adopted for all reported experiments. Batch size 1 was necessitated by the high spatial resolution of the structural MRI volumes ($197\times 233\times 189$ voxels): loading all four branches (two functional, two structural) simultaneously at a larger batch size was infeasible within the 24 GB memory budget of the available GPU. Batch normalization was nevertheless retained for architectural consistency across all four branches, and the resulting instability of per-sample statistics is discussed explicitly as a limitation below rather than left unacknowledged.

### C. Leave-One-Site-Out (LOSO) Cross-Validation

Because the shuffled stratified 5-fold protocol above does not stratify by acquisition site, same-site subjects can appear on both sides of a split, and the 74.80% figure alone cannot be read as evidence of cross-site generalization. To address this, we additionally evaluated the model under Leave-One-Site-Out (LOSO) cross-validation: for each of the seven sites in turn, the model was trained from scratch on the remaining six sites and evaluated on the held-out site in full, so that no subject from the test site was seen during training under any circumstance. Table VI reports the resulting per-site accuracy.

Restricted to the five sites where both diagnostic groups are present (KKI, NeuroIMAGE, NYU, OHSU, Peking; *N=620*), per-site LOSO accuracy ranges from 73.40% (OHSU) to 79.74% (NYU), with an unweighted mean of **76.54% *±* 2.41%** and a subject-weighted (pooled) mean of 76.96%. Across all seven sites, including the two TDC-only sites, the unweighted mean is 74.94% *±* 3.25% and the subject-weighted mean is 75.79%. In every case, LOSO accuracy is comparable to, and on the mixed-diagnosis-site subset numerically higher than, the within-distribution 5-fold figure (74.80%), which is the opposite of what a model relying primarily on site-specific signal would be expected to show: such a model's performance should degrade, not hold steady or improve, when the entire held-out site was never seen during training. We read this as reasonably supportive evidence against the model relying primarily on site-specific signal, while stopping short of treating it as a definitive generalization guarantee: LOSO accuracy still varies by more than 6 percentage points across the five mixed-diagnosis sites (73.40%–79.74%), and the two TDC-only sites cannot be used to assess sensitivity at all, since the ADHD-200 release provides no ADHD-labelled subject at either site. Both the shuffled 5-fold result and the LOSO result are reported henceforth; the LOSO result is treated as the primary evidence for cross-site behavior, and the shuffled 5-fold result as a secondary, within-distribution reference.

### D. Baseline Comparison

Multi-model 1 is a time-distributed architecture that accepts the raw 4D rs-fMRI volume alongside the resized 3D sMRI. The fMRI branch uses three Conv3D–Pool–BN blocks with a top-down filter progression (256, 128, 64), followed by a Gated Recurrent Unit (GRU) recurrent layer of 32 units that aggregates temporal dynamics across the volume. sMRI passes through four convolutional layers with filters of sizes 64, 64, 128, and 256. Both streams are concatenated into a 128-d representation before the sigmoid output. The model was trained with Adam at $10^{-3}$ and a batch size of 2 for 100 epochs using binary cross-entropy loss. This baseline achieved a validation accuracy of 70.64% on a single split across six sites (Table II). Despite the GRU's ability to model temporal dependencies, the spatial compression introduced by trilinear resizing and the absence of modality-specific branches limit its discriminative capacity relative to the proposed model.

### E. Comparison With Voxel-Level State-of-The-Art

Table VII compares the proposed model exclusively against methods that use the ADHD-200 Athena-preprocessed dataset with 3D volumetric voxel-level inputs, matching the exact experimental conditions of the present work. All listed methods avoid ROI-averaged FC features and instead operate on the same pre-computed 3D derivative maps (fALFF, ReHo, GM, WM) available from the Athena pipeline. We also discuss one recent (2026) voxel-level method, VoxSTNet [29] – disclosed here as prior work by the same author team as the present manuscript, rather than an independent third-party comparison – which is deliberately *not* included as a row in Table VII: unlike every method listed there, VoxSTNet operates directly on raw 4D rs-fMRI BOLD volumes rather than on Athena's pre-computed derivative maps, so it does not meet this table's own matched-input inclusion criterion, and a direct row-for-row comparison would conflate two different data regimes regardless of shared authorship. We discuss it narratively instead, immediately below the table.

*vs. Zou et al. [2]:* The foundational voxel-level 3D-CNN study. It tests six individual derivatives and evaluates pairwise multimodal combinations; the best pair (fALFF+GM) achieves 69.15% on a single hold-out split. The proposed model extends this to all four relevant derivatives (fALFF, ReHo, GM, WM) and jointly processes them through dedicated branches with hierarchical fusion, achieving **+5.65 pp** with a more statistically reliable 5-fold CV.

*vs. Wang et al. [3]:* Uses only GM density from sMRI with a dilated 3D-CNN to enlarge the receptive field. The single-modality structural approach achieves approximately 63%, confirming that sMRI alone is insufficient and that functional derivatives (fALFF, ReHo) are essential complements.

*vs. Vuyyuru et al. (VoxSTNet, 2026) [29]:* VoxSTNet and the present work differ along three axes simultaneously, and we are explicit about all three rather than treating them as one difference: (i) *data regime* – VoxSTNet processes raw, minimally processed 4D rs-fMRI BOLD volumes directly from the initial ADHD-200 release, whereas the present work consumes only the Athena pipeline's pre-computed derivative volumes (fALFF, ReHo, GM, WM); (ii) *modality count* – VoxSTNet is a single-modality system operating on rs-fMRI alone, with no structural MRI input or fusion stage, whereas the present work is explicitly multimodal, fusing two functional derivatives (fALFF, ReHo) with two structural derivatives (GM, WM) through a dedicated four-branch, two-stage hierarchical fusion architecture; and (iii) *network family* – VoxSTNet is a spatio-temporal network designed to model the full BOLD time series directly, whereas the present work is a set of independent 3D-CNN branches over static, already-time-collapsed derivative volumes, with no temporal modeling component at all. Given these three simultaneous differences, VoxSTNet's reported shuffled 5-fold accuracy ($98.7\%\pm 0.4\%$) is not a meaningful point of comparison against our own 5-fold result: it reflects a different dataset regime, a different (single) modality count, and a different network family all at once, and none of those differences are attributable to the two-stage hierarchical fusion contribution this paper is evaluating. Their Leave-One-Site-Out accuracy (78.4%, 95% CI: 75.1–81.7%) is the one figure we consider a meaningful reference point, since it is evaluated under the same site-generalization logic as our own LOSO result in the present work (Section III-B): our mean LOSO accuracy on the five mixed-diagnosis sites (76.54%) is close to, though somewhat below, this earlier reported LOSO figure for the raw-input, single-modality system. We read this as indicating that, across our own two lines of work, raw single-modality modeling and derivative-based multimodal modeling are broadly comparable in cross-site robustness on this cohort, with any remaining gap a reasonable target for future integration of the two approaches rather than evidence that one is categorically superior to the other.

In summary, the proposed four-source hierarchical architecture is the only method in this comparison that (i) jointly processes all four Athena-derived 3D voxel maps through dedicated branches, (ii) applies a two-stage hierarchical fusion reflecting the functional/structural divide, and (iii) evaluates on a large pooled multi-site dataset (768 subjects, 7 sites) with 5-fold CV, yielding a robust and reproducible accuracy of **74.80%** (*±*0.47%).

## IV. Discussion

A systematic ablation was conducted to decompose the performance gain into contributions from individual modalities and from the hierarchical fusion topology. Thirteen model variants were trained and evaluated under the same protocol as the full model: four single-branch variants, three pairwise combinations, four three-branch variants, each excluding one derivative, a flat-concatenation four-branch variant, and the proposed hierarchical model. Branch architectures, optimizer settings (Adam, $\alpha \!=\!10^{-3}$, batch size 1, 100-epoch ceiling, best-checkpoint selection), and evaluation splits were held constant across all variants; only the active modality channels and the fusion stage were varied. Results appear in Table VIII.

*a) Single-branch analysis:* Among individual modalities, fALFF yields the highest accuracy (68.70% *±* 0.50%, independently reconfirmed–see table footnote), consistent with its established sensitivity to prefrontal-striatal BOLD dysregulation in ADHD; ReHo follows (67.83%), confirming the discriminative value of local BOLD synchrony. Both structural maps underperform the functional derivatives (GM: 65.58%; WM: 63.96%, the weakest single predictor), reflecting that axonal abnormalities in ADHD are more diffuse and spatially subtle than grey-matter density differences. All four modalities individually exceed chance (50%), confirming an independent diagnostic signal per modality. Each single-branch row uses the same architectural design (block depth, filter schedule, embedding width) as its corresponding branch inside the full model, so differences reflect input information rather than uneven network capacity.

*b) Capacity-Matched Single-Modality Analysis:* To determine directly whether the observed performance improvement is attributable to increased model capacity rather than multimodal fusion, four capacity-matched single-modality networks were additionally constructed, one per derivative (ReHo, fALFF, GM, WM), each independently widened/deepened so that its trainable parameter count closely matches the proposed hierarchical fusion network. Table IX reports the results.

**TABLE IX:** Capacity-Matched Single-Modality Control Models Vs. the Proposed Hierarchical Fusion Network. Parameter Counts Span a Range of Only 0.60% (683,912–688,044), Well Within the Commonly Used *±*5% Criterion for Capacity-Matched Comparisons

**Model**	**Params.**	**Val. Acc. (%)**
Capacity-matched ReHo	683,912	63.70
Capacity-matched fALFF	684,110	64.90
Capacity-matched GM	688,044	61.80
Capacity-matched WM	685,996	62.60
**Proposed (hier. fusion)**	**686,817**	**74.80**

Despite parameter counts matched to within 0.60% of the proposed network, none of the four capacity-matched single-modality models approaches the fused model's accuracy: the best (capacity-matched fALFF, 64.90%) trails by 9.90 pp, and the four-model average (63.25%) trails by 11.55 pp–comparable to the single-modality gaps in Table VIII above (9.90–13.00 pp), now obtained under matched rather than merely consistent capacity. This rules out increased model capacity as the source of the proposed model's advantage, attributing the improvement instead to hierarchical integration of complementary structural and functional information.

*c) Pairwise analysis:* The fALFF + ReHo pair (71.43%) outperforms the structural pair GM + WM (69.02%), indicating that functional derivatives collectively carry more ADHD-discriminative information than structural maps alone. The fALFF + GM pair (69.74%) replicates and marginally improves upon the best result of Zou et al. [2] (69.15%) under identical preprocessing, validating the comparability of our experimental conditions.

*d) Three-branch analysis:* All four three-branch configurations exceed the best pairwise result, confirming that every modality contributes incremental value beyond any subset of three. Dropping WM yields the smallest reduction in accuracy relative to the full model (*-1.94* pp), whereas dropping fALFF yields the largest (*-2.66* pp), corroborating the central role of this functional derivative. Importantly, no single modality can be removed without degrading all four metrics simultaneously.

*e) Fusion strategy:* Replacing the two-stage hierarchical fusion with flat concatenation of all four branch embeddings (73.51%) degrades accuracy by 1.29 pp, confirming that explicitly encoding the functional/structural divide–merging within-modality pairs before combining across modalities–extracts complementary inter-modality structure that flat concatenation conflates, independent of the four-branch input strategy.

*f) Systematic Fusion-Strategy Ablation:* Beyond flat concatenation, we evaluated three further fusion strategies under the identical protocol, architectures, and splits, isolating the fusion mechanism alone: (i) *pairwise fusion*, an alternative, non functional/structural grouping (e.g., ReHo+GM and fALFF+WM); (ii) *late fusion*, averaging four independent single-modality classifiers' output probabilities with no feature-level concatenation; and (iii) *attention-based fusion*, replacing concatenation with a learned attention mechanism over the four embeddings. Table X reports the results.

**TABLE X:** Fusion-Strategy Ablation: Five Alternative Ways of Combining the Four Modality Branches, Evaluated Under the Identical Training Protocol

**Fusion Strategy**	**Description**	**Val. Acc. (%)**
Flat concatenation	All four embeddings concatenated directly	73.51
Pairwise fusion	Alternative modality pairing (e.g., ReHo+GM, fALFF+WM)	66.70
Late fusion	Average of four independent single-modality classifiers' output probabilities	68.45
Attention-based fusion	Learnable attention weights across the four modality embeddings	65.40
**Hierarchical fusion (proposed)**	**Functional pair and structural pair fused separately, then combined**	**74.80**

The proposed hierarchical fusion outperforms every alternative, by margins from 1.29 pp (flat concatenation) to 9.40 pp (attention-based fusion). Pairwise fusion under an alternative, non functional/structural grouping underperforms both flat concatenation and the proposed design, indicating that which modalities are paired measurably matters: the functional/structural split reflects genuine shared structure (ReHo and fALFF both from rs-fMRI; GM and WM both from sMRI) that an alternative pairing does not capture. Attention-based fusion, despite being the most parametrically flexible alternative, performs *worst* overall–consistent with a learned attention mechanism overfitting on a cohort of this size (*N=768*), since it adds trainable parameters without a corresponding increase in training data. Such mechanisms remain worth revisiting with a larger cohort or stronger regularization, but are not, on present evidence, an improvement over the simpler hierarchical design.

*Limitations:* Several limitations should be acknowledged. First, the study relies on ADHD-200 Athena-preprocessed derivatives; future work should validate sensitivity in independently processed cohorts. Second, LOSO cross-validation (Section III-B) shows per-site accuracy on the five mixed-diagnosis sites from 73.40% to 79.74%, comparable to or exceeding the within-distribution 5-fold figure–reassuring but not a definitive generalization guarantee, since the two TDC-only sites (Pittsburgh, WashU) cannot be used to assess sensitivity, age/sex confounds are statistically detectable (see below), and a *>6* pp spread remains across sites. Third, the model omits temporal dynamics from the raw BOLD series, which may carry complementary information beyond the static fALFF/ReHo maps. Fourth, we quantified age/sex confounds using the official ADHD-200 phenotypic release in two complementary ways. In a subject-matched 5-site subset (KKI, NeuroIMAGE, OHSU, Pittsburgh, WashU; *n=358* of 768, 46.6% of the cohort–NYU and Peking could not be linked due to a differing identifier scheme), TDC subjects are slightly but significantly older than ADHD subjects (mean *12.51± 3.81* vs. * 11.51± 3.92* years; Welch's *t=2.05*, *p=0.042*), and sex distribution differs (51.1% vs. 69.0% male; $\chi ^{2}=7.67$, *p=0.0056*). A broader, unmatched check across all seven sites (*n=577*; TDC and ADHD-Combined categories only) shows the same pattern more strongly (mean *12.22± 3.46* vs. * 11.01± 3.18* years, *t=3.83*, *p< 0.001*; 47.2% vs. 79.4% male, $\chi ^{2}=44.66$, *p< 0.001*), indicating these are genuine, robustly detectable confounds rather than an artifact of either analysis's scope. No covariate regression, age/sex matching, or motion-based adjustment was performed; motion metrics (framewise displacement, DVARS, RMS) were unavailable in the phenotypic files obtained. These demographic differences are inherent to the public ADHD-200 dataset rather than an artifact of the proposed framework. A covariate-aware variant–concatenating age/sex alongside the branch embeddings and re-validating the full CV/LOSO protocols to confirm it does not merely exploit demographic imbalance–constitutes dedicated future work rather than an incremental change. Finally, BatchNorm with batch size 1 may yield unstable per-sample statistics under site-imbalanced mini-batches; InstanceNorm3d or gradient accumulation is recommended as a future refinement.

### A. Clinical Translation Considerations

This section sets out what would be required before a system of this kind could play any role in clinical care.

*Decision support, not replacement:* This pipeline is a research prototype demonstrating that voxel-level, multi-derivative fusion is a viable direction for imaging-based ADHD classification, not a diagnostic instrument. ADHD diagnosis should remain a clinical judgment informed by structured interviews, behavioral rating scales, and longitudinal observation; any future clinical role would be as a decision-support aid alongside, not instead of, standard assessment.

*Prospective and external validation:* The results here, including LOSO, are retrospective analyses of a single public dataset (ADHD-200). Clinical translation would require prospective validation on independently recruited cohorts, scanned on hardware/protocols not represented in training and reflecting the deployment population's diversity–retrospective accuracy on a well-studied benchmark is necessary but insufficient for clinical use.

*Regulatory approval:* Any diagnostic support role would require evaluation as software as a medical device (e.g., under FDA or equivalent frameworks), including intended-use documentation, clinical validation studies, and risk classification. No such process has been initiated, and no result here should be read as evidence of regulatory readiness.

*Explainability as a precondition, not a feature:* The interpretability limitations discussed above (uncertain confound control, unresolved site-generalization variability) are blocking issues for clinical consideration, not optional enhancements–a model whose failure modes are not characterized across demographic and site subgroups is not appropriate for deployment regardless of aggregate accuracy.

*External validation and scanner harmonization:* ADHD-200 spans seven sites with heterogeneous field strengths, repetition times, and voxel resolutions (Table I), a strength for the generalization analysis here but no guarantee of performance on scanners or populations outside this consortium. Deployment would need explicit harmonization (e.g., ComBat-style correction) and validation spanning the target clinical sites.

In summary, this work is a methodological contribution–a multimodal, hierarchical fusion architecture that outperforms flat concatenation and single-modality baselines with encouraging cross-site LOSO evidence–rather than a clinical instrument, and we view stating this distinction explicitly as part of responsible reporting for imaging-based mental health classifiers.

## V. Conclusion

A four-branch voxel-level 3D-CNN with two-stage hierarchical fusion was developed for ADHD classification using the ADHD-200 Athena dataset. The core design principle is to preserve modality-specific spatial statistics: rather than merging all inputs through shared weights or flattening them into ROI connectivity vectors, each of the four Athena derivatives—fALFF, ReHo, GM, and WM—is assigned a dedicated branch whose depth and filter schedule are calibrated to its native resolution. Fusion proceeds in two structured stages: functional embeddings are consolidated first, structural embeddings second, and the two 64-d summaries are subsequently combined into a 128-d joint representation for prediction. Under 5-fold cross-validation across all 768 participants from seven acquisition sites, the proposed framework attained $74.80\%\,\pm \,0.47\%$ accuracy—a 5.65 percentage-point improvement over the strongest comparable voxel-level baseline. The narrow inter-fold spread confirms that performance gains are consistent across partitions rather than attributable to a single advantageous split. Because this shuffled 5-fold protocol does not stratify by site, we additionally evaluated the model under Leave-One-Site-Out cross-validation: on the five sites containing both diagnostic groups, per-site accuracy ranged from 73.40% to 79.74% (mean $76.54\%\,\pm \,2.41\%$), comparable to or exceeding the within-distribution figure and providing reassuring, though not definitive, evidence against the model relying primarily on site-specific signal. These results demonstrate that modality-specific encoding and structurally motivated fusion jointly account for the observed improvement and that neither component alone is sufficient to replicate the full gain. All experimentation reported here uses the standard, publicly available ADHD-200 consortium dataset rather than independently or locally acquired scans: prospectively recruiting and scanning an ADHD cohort requires clinical partnerships, ethics approval, and dedicated scanner time that are beyond the scope of a single modeling study, whereas the ADHD-200 release provides a large, multi-site cohort suitable for the voxel-level, multi-derivative approach developed here. Model training was performed on locally acquired hardware (an NVIDIA RTX 4090 GPU; Section III-B). Planned extensions include attention-weighted cross-modal fusion, an explicit motion- and age-confound analysis, matched-protocol reimplementation of key literature baselines, adaptation to Autism Spectrum Disorder (ASD) classification using the same Athena-derived derivative maps, and, as a longer-term extension beyond the standard-dataset experiments in this paper, prospective collection of real-time ADHD neuroimaging data to complement and further validate the findings reported here. Readers seeking the full per-branch layer specifications, the complete symbolic derivation of the forward pass, and the generic multi-model training loop underlying Section II-C are directed to the Supplementary Materials, available online, which reproduce this technical detail in full without repeating it in the main text.

## Supplementary Materials

Supplementary Materials
