# Supplementary material for: Four-Source Voxel-Level 3D-CNN With Hierarchical Feature Fusion of rs-fMRI and sMRI Derivatives for ADHD Classification
Source: IEEE Open J Eng Med Biol. 2026 Sep 9;7:341–54. doi: 10.1109/OJEMB.2026.3732708 (PMC13667363; doi:10.1109/OJEMB.2026.3732708)
Supplement: Supplementary file 1 [file supp1-3732708.pdf]

Supplementary Materials

A. Extended Architecture Tables

Tables SI and SII give the complete per-layer specification of the four branches–input/output shapes, kernel and pooling sizes, and per-layer filter counts–summarized architecturally in Section II-C.

B. Full Symbolic Derivation of the Forward Pass

1) Symbolic system and operators: We model each modality as a 3D signal (depth, height, width) and define a small family of operators that act on 3D tensors.

$$\mathbf{X}_A, \mathbf{X}_B \in \mathbb{R}^{49\times58\times47\times1} \qquad \text{(ReHo, fALFF)}$$
$$\mathbf{X}_C, \mathbf{X}_D \in \mathbb{R}^{197\times233\times189\times1} \qquad \text{(GM, WM)}$$

Compact operators (all convolutions use *valid* padding):

$$\begin{aligned}
\mathcal{C}_{k,c}[\cdot]&: \text{ 3-D conv., } k^3 \text{ kernel, } c \text{ channels}\\
\mathcal{P}[\cdot]&: \text{ 3-D max-pool, factor \& stride } (2,2,2)\\
\mathcal{B}[\cdot]&: \text{ Batch Norm (affine, } \gamma,\beta \text{ learnable)}\\
\mathcal{D}_p[\cdot]&: \text{ Dropout, rate } p \text{ (train only)}\\
\mathcal{F}[\cdot]&: \text{ Flatten to 1-D vector}\\
\mathcal{L}_u[\cdot]&: \text{ Dense layer, } u \text{ outputs}\\
\varphi(\cdot)&: \text{ ReLU;} \quad \sigma(\cdot): \text{ sigmoid}\\
[\,;\,]&: \text{ vector concatenation}
\end{aligned}$$

2) Branch processing–operational form: We define each branch as a sequential application of the operator pipeline. The notation below is compact; apply operators left to right.

a) ReHo (branch A) & fALFF (branch B)::

$$\begin{aligned}
\mathbf{T}^{(0)} &:= \mathbf{X}_s, \qquad s \in \{A, B\}\\
\mathbf{T}^{(1)} &:= \mathcal{D}_{0.5}\Big(\mathcal{B}\big(\mathcal{P}(\varphi(\mathcal{C}_{3,16}[\mathbf{T}^{(0)}]))\big)\Big)\\
\mathbf{T}^{(2)} &:= \mathcal{D}_{0.5}\Big(\mathcal{B}\big(\mathcal{P}(\varphi(\mathcal{C}_{3,32}[\mathbf{T}^{(1)}]))\big)\Big)\\
\mathbf{T}^{(3)} &:= \mathcal{B}\big(\mathcal{P}(\varphi(\mathcal{C}_{3,64}[\mathbf{T}^{(2)}]))\big)\\
\mathbf{z}_s &:= \mathcal{F}[\mathbf{T}^{(3)}] \in \mathbb{R}^{5120}\\
\mathbf{h}_s &:= \varphi\big(\mathcal{L}_{32}[\mathbf{z}_s]\big) \in \mathbb{R}^{32}.
\end{aligned}$$

The concrete spatial sizes (verified from the implementation) evolve as:

$$(49,58,47) \xrightarrow{\mathcal{C}_{3,16}} (47,56,45) \xrightarrow{\mathcal{P}} (23,28,22) \xrightarrow{\mathcal{C}_{3,32}} (21,26,20) \xrightarrow{\mathcal{P}} (10,13,10) \xrightarrow{\mathcal{C}_{3,64}} (8,11,8) \xrightarrow{\mathcal{P}} (4,5,4)$$

so $|\mathbf{z}_s| = 4\cdot5\cdot4\cdot64 = 5120$.

b) GM (branch C) & WM (branch D)::

$$\begin{aligned}
\mathbf{U}^{(0)} &:= \mathbf{X}_t, \qquad t \in \{C, D\}\\
\mathbf{U}^{(1)} &:= \mathcal{D}_{0.5}\Big(\mathcal{B}\big(\mathcal{P}(\varphi(\mathcal{C}_{3,16}[\mathbf{U}^{(0)}]))\big)\Big)\\
\mathbf{U}^{(2)} &:= \mathcal{D}_{0.5}\Big(\mathcal{B}\big(\mathcal{P}(\varphi(\mathcal{C}_{3,16}[\mathbf{U}^{(1)}]))\big)\Big)\\
\mathbf{U}^{(3)} &:= \mathcal{D}_{0.5}\Big(\mathcal{B}\big(\mathcal{P}(\varphi(\mathcal{C}_{3,16}[\mathbf{U}^{(2)}]))\big)\Big)\\
\mathbf{U}^{(4)} &:= \mathcal{D}_{0.5}\Big(\mathcal{B}\big(\mathcal{P}(\varphi(\mathcal{C}_{3,16}[\mathbf{U}^{(3)}]))\big)\Big)\\
\mathbf{U}^{(5)} &:= \mathcal{B}\big(\mathcal{P}(\varphi(\mathcal{C}_{3,32}[\mathbf{U}^{(4)}]))\big)\\
\mathbf{z}_t &:= \mathcal{F}[\mathbf{U}^{(5)}] \in \mathbb{R}^{1920}\\
\mathbf{h}_t &:= \varphi\big(\mathcal{L}_{32}[\mathbf{z}_t]\big) \in \mathbb{R}^{32}.
\end{aligned}$$

The spatial shape progression here is:

$$(197,233,189) \xrightarrow{\mathcal{C}_3} (195,231,187) \xrightarrow{\mathcal{P}} (97,115,93) \xrightarrow{\mathcal{C}_3} (95,113,91) \xrightarrow{\mathcal{P}} (47,56,45) \xrightarrow{\mathcal{C}_3} (45,54,43) \xrightarrow{\mathcal{P}} (22,27,21) \xrightarrow{\mathcal{C}_3} (20,25,19) \xrightarrow{\mathcal{P}} (10,12,9) \xrightarrow{\mathcal{C}_3} (8,10,7) \xrightarrow{\mathcal{P}} (4,5,3)$$

hence $|\mathbf{z}_t| = 4\cdot5\cdot3\cdot32 = 1920$.

3) Modal fusion as structured concatenation: We group and compress the 4 branch encodings:

$$\mathbf{u}_{\text{fMRI}} := [\mathbf{h}_A; \mathbf{h}_B] \in \mathbb{R}^{64}, \quad \mathbf{u}_{\text{MRI}} := [\mathbf{h}_C; \mathbf{h}_D] \in \mathbb{R}^{64}. \tag{1}$$

Apply two independent projection heads (dense + ReLU) and then fuse:

$$\mathbf{p}_{\text{fMRI}} := \varphi\big(\mathcal{L}_{64}[\mathbf{u}_{\text{fMRI}}]\big), \quad \mathbf{p}_{\text{MRI}} := \varphi\big(\mathcal{L}_{64}[\mathbf{u}_{\text{MRI}}]\big). \tag{2}$$

Concatenate and classify:

$$\mathbf{q} := [\mathbf{p}_{\text{fMRI}}; \mathbf{p}_{\text{MRI}}] \in \mathbb{R}^{128}, \quad \mathbf{v} := \varphi\big(\mathcal{L}_{128}[\mathbf{q}]\big) \in \mathbb{R}^{128}, \tag{3}$$

$$\hat{y} = \sigma\big(\mathcal{L}_1[\mathbf{v}]\big) \in (0,1). \tag{4}$$

4) Parameter accounting (selected exact terms): To make the model reproducible, we list key parameter counts (explicit arithmetic).

a) Dense layers (exact):

$$\begin{aligned}
\#\text{params}(\mathcal{L}_{32}, \text{ReHo}) &= 5120\cdot32 + 32 = 163{,}872\\
\#\text{params}(\mathcal{L}_{32}, \text{fALFF}) &= 5120\cdot32 + 32 = 163{,}872\\
\#\text{params}(\mathcal{L}_{32}, \text{GM}) &= 1920\cdot32 + 32 = 61{,}472\\
\#\text{params}(\mathcal{L}_{32}, \text{WM}) &= 1920\cdot32 + 32 = 61{,}472
\end{aligned}$$

b) Fusion/classifier (exact):

$$\begin{aligned}
\#\text{params}(\mathcal{L}_{64}) &= 64\cdot64 + 64 = 4{,}160 \ (\times2)\\
\#\text{params}(\mathcal{L}_{128}) &= 128\cdot128 + 128 = 16{,}512\\
\#\text{params}(\mathcal{L}_{1}) &= 128\cdot1 + 1 = 129
\end{aligned}$$

c) Conv / BN examples (illustrative): The Conv3D parameter count is:

$$\#\text{params}(\mathcal{C}_{3,c}, C_{\text{in}}) = 3^3\cdot C_{\text{in}}\cdot c + c.$$

First conv (ReHo/fALFF), $C_{\text{in}}=1$, $c=16$:

$$\# = 27\cdot1\cdot16 + 16 = 448.$$

TABLE SI. Architecture summary for the four-branch proposed model. BN = Batch Normalisation; MP = MaxPool3D (2^3, stride 2^3); FC = Dense (fully connected); Drp = Dropout(0.5).

Pipeline	L#	Operation	Input Shape	Output Shape	Ker./Pool	Notes
ReHo (Branch A) fALFF (Branch B)	1	Conv3D	$49 \times 58 \times 47 \times 1$	$47 \times 56 \times 45 \times 16$	3^3/—	ReLU, BN
	2	MP	$47 \times 56 \times 45 \times 16$	$23 \times 28 \times 22 \times 16$	—/2^3	—
	3	Conv3D	$23 \times 28 \times 22 \times 16$	$21 \times 26 \times 20 \times 32$	3^3/—	ReLU, BN, Drp
	4	MP	$21 \times 26 \times 20 \times 32$	$10 \times 13 \times 10 \times 32$	—/2^3	—
	5	Conv3D	$10 \times 13 \times 10 \times 32$	$8 \times 11 \times 8 \times 64$	3^3/—	ReLU, BN
	6	MP + Flatten	$8 \times 11 \times 8 \times 64$	5120	—/2^3	—
	7	FC	5120	32	—	ReLU
GM (Branch C) WM (Branch D)	1	Conv3D	$197 \times 233 \times 189 \times 1$	$195 \times 231 \times 187 \times 16$	3^3/—	ReLU, BN
	2	MP	$195 \times 231 \times 187 \times 16$	$97 \times 115 \times 93 \times 16$	—/2^3	—
	3	Conv3D	$97 \times 115 \times 93 \times 16$	$95 \times 113 \times 91 \times 16$	3^3/—	ReLU, BN, Drp
	4	MP	$95 \times 113 \times 91 \times 16$	$47 \times 56 \times 45 \times 16$	—/2^3	—
	5	Conv3D	$47 \times 56 \times 45 \times 16$	$45 \times 54 \times 43 \times 16$	3^3/—	ReLU, BN, Drp
	6	MP	$45 \times 54 \times 43 \times 16$	$22 \times 27 \times 21 \times 16$	—/2^3	—
	7	Conv3D	$22 \times 27 \times 21 \times 16$	$20 \times 25 \times 19 \times 16$	3^3/—	ReLU, BN
	8	MP+Conv3D	$20 \times 25 \times 19 \times 16$	1920	3^3/2^3	ReLU, BN, Drp
	9	Flatten + FC	1920	32	—	ReLU
Fusion	F1	Concat+FC	$[h_A; h_B] \in \mathbb{R}^{64}$	$\mathbb{R}^{64}$	—	ReLU
	F2	Concat+FC	$[h_C; h_D] \in \mathbb{R}^{64}$	$\mathbb{R}^{64}$	—	ReLU
	F3	Concat+FC	$[p_{\text{fMRI}}; p_{\text{MRI}}] \in \mathbb{R}^{128}$	$\mathbb{R}^{128}$	—	ReLU
	F4	FC (output)	$\mathbb{R}^{128}$	$\mathbb{R}^{1}$	—	Sigmoid

TABLE SII. Detailed architecture of the four independent pipelines (ReHo, fALFF, GM, WM).

Layer	ReHo Branch (A)	fALFF Branch (B)	GM Branch (C)	WM Branch (D)
Input Shape	$49 \times 58 \times 47 \times 1$	$49 \times 58 \times 47 \times 1$	$197 \times 233 \times 189 \times 1$	$197 \times 233 \times 189 \times 1$
Conv3D_1	16 filters (8×2), 3^3, ReLU	16 filters (8×2), 3^3, ReLU	16 filters (4×4), 3^3, ReLU	16 filters (4×4), 3^3, ReLU
MaxPool3D_1	2^3, stride 2^3	2^3, stride 2^3	2^3, stride 2^3	2^3, stride 2^3
BatchNorm_1	Yes	Yes	Yes	Yes
Conv3D_2	32 filters (8×4), 3^3, ReLU	32 filters (8×4), 3^3, ReLU	16 filters (4×4), 3^3, ReLU	16 filters (4×4), 3^3, ReLU
MaxPool3D_2	2^3, stride 2^3	2^3, stride 2^3	2^3, stride 2^3	2^3, stride 2^3
BatchNorm_2 / Dropout	BN, Dropout(0.5)	BN, Dropout(0.5)	BN, Dropout(0.5)	BN, Dropout(0.5)
Conv3D_3	64 filters (8×8), 3^3, ReLU	64 filters (8×8), 3^3, ReLU	16 filters (4×4), 3^3, ReLU	16 filters (4×4), 3^3, ReLU
MaxPool3D_3	2^3, stride 2^3	2^3, stride 2^3	2^3, stride 2^3	2^3, stride 2^3
BatchNorm_3	Yes	Yes	BN, Dropout(0.5)	BN, Dropout(0.5)
Conv3D_4 (GM/WM only)	—	—	16 filters (4×4), 3^3, ReLU	16 filters (4×4), 3^3, ReLU
MaxPool3D_4 / BN (GM/WM)	—	—	2^3, BN, Dropout(0.5)	2^3, BN, Dropout(0.5)
Conv3D_5 (GM/WM only)	—	—	32 filters (4×8), 3^3, ReLU	32 filters (4×8), 3^3, ReLU
MaxPool3D_5 / BN (GM/WM)	—	—	2^3, BN, Dropout(0.5)	2^3, BN, Dropout(0.5)
Flatten	Yes	Yes	Yes	Yes
Dense_out	32 units, ReLU	32 units, ReLU	32 units, ReLU	32 units, ReLU
**Flat. vector size**	$4 \times 5 \times 4 \times 64 = 5120$	5120	$4 \times 5 \times 3 \times 32 = 1920$	1920

BatchNorm on a C-channel tensor contributes $2C$ trainable parameters (scale + shift).

5) Global totals (from implementation): Summing the layerwise terms as in the Keras `model.summary()` gives the overall counts

$$\begin{aligned} \text{Total params} &= 686{,}817 \\ \text{Trainable} &= 685{,}985 \\ \text{Non-trainable} &= 832 \end{aligned}$$

6) Complete forward map: The full forward pass of the proposed model, from four input maps to a binary prediction, can be written concisely as a composition of the operators defined in Section B-1. Let $\Phi_s(\mathbf{X}_s)$ denote the branch encoder for input $s \in \{A, B, C, D\}$:

$$\Phi_s(\mathbf{X}_s) = \varphi(\mathcal{L}_{32}(\mathcal{F}(\mathcal{B}(\mathcal{P}(\varphi(\mathcal{C}[\mathbf{X}_s])))))) \in \mathbb{R}^{32}$$

where $\mathcal{C}[\cdot]$ represents the full stack of Conv3D–Pool–BN blocks specific to each branch (3 blocks for functional branches A, B; 5 blocks for structural branches C, D). The two-stage hierarchical fusion and final classification are then:

$$\begin{aligned} \mathbf{p}_{\text{fMRI}} &= \varphi(\mathcal{L}_{64}([\Phi_A; \Phi_B])) \in \mathbb{R}^{64} \\ \mathbf{p}_{\text{MRI}} &= \varphi(\mathcal{L}_{64}([\Phi_C; \Phi_D])) \in \mathbb{R}^{64} \\ \mathbf{v} &= \varphi(\mathcal{L}_{128}([\mathbf{p}_{\text{fMRI}}; \mathbf{p}_{\text{MRI}}])) \in \mathbb{R}^{128} \\ \hat{y} &= \sigma(\mathcal{L}_1(\mathbf{v})) \in (0, 1) \end{aligned}$$

The complete end-to-end map is therefore:

$$\hat{y} = \sigma(\mathcal{L}_1(\varphi(\mathcal{L}_{128}([\,\varphi(\mathcal{L}_{64}([\Phi_A; \Phi_B]))\ ;\ \varphi(\mathcal{L}_{64}([\Phi_C; \Phi_D]))\,])))) \tag{5}$$

where $[\,;\,]$ denotes vector concatenation and all φ are ReLU activations.

Algorithm S1 Algorithm for Four-Source Voxel-Level 3D-CNN with Hierarchical Feature Fusion

Require: Training data X_{train}, Validation data X_{val}
Require: Hyperparameters: epochs E, batch size B, learning rate η
for each epoch e from 1 **to** E **do**
  for each batch b in training data **do**
    Sample batch: fALFF, ReHo from rs-fMRI and GM, WM from MRI, with labels $Y_{\text{batch}}^{(b)}$
    Preprocess each modality separately: $X_{\text{pre}}^{(b)} = \text{preprocess}(X_{\text{batch}}^{(b)})$
    Compute binary cross-entropy loss: $\mathcal{L}^{(b)} = -\frac{1}{B}\sum_{i=1}^{B}[y_i^{(b)} \log \hat{y}_i^{(b)} + (1-y_i^{(b)}) \log(1-\hat{y}_i^{(b)})]$
    Compute gradients: $\nabla_\theta \mathcal{L}^{(b)}$
    Update parameters: $\theta \leftarrow \theta - \eta \nabla_\theta \mathcal{L}^{(b)}$
  end for
  for each batch b' in validation data **do**
    Sample validation batch $X_{\text{batch}}^{(b')}$ with labels $Y_{\text{batch}}^{(b')}$
    Preprocess: $X_{\text{pre}}^{(b')} = \text{preprocess}(X_{\text{batch}}^{(b')})$
    Compute validation loss and evaluation metrics on X_{val}
  end for
  Log training and validation metrics for epoch e
end for

7) Training objective: Optimize parameters Θ by minimizing the binary cross-entropy:

$$\mathcal{L}(\Theta) = -\frac{1}{N}\sum_{i=1}^{N}\left[y_i \log \hat{y}_i + (1-y_i)\log(1-\hat{y}_i)\right] + \lambda\|\Theta\|_2^2, \quad \lambda = 0 \text{ in the reported experiments} \tag{6}$$

trained with Adam (no explicit weight decay or per-layer kernel regularizer was applied) for 100 epochs and batch size 1 (as in the implementation).

C. Generic Multimodal Training and Validation Loop

Algorithm S1 states the generic multimodal training and validation loop shared conceptually across all model variants examined in this paper (including the Multi-model 1/2 baselines of Section III-D), which Algorithm **??** in Section II-C specializes to the proposed four-branch hierarchical model.
